# Case report: Treatment of joint supplement toxicity resulting in acidemia, hyperglycemia, electrolyte derangements, and multiple organ dysfunction

**DOI:** 10.3389/fvets.2023.1141978

**Published:** 2023-06-29

**Authors:** Nicole M. Bunnell, Linda Weatherton

**Affiliations:** Department of Emergency and Critical Care, Veterinary Emergency and Critical Care, Las Vegas, NV, United States

**Keywords:** joint supplement, hypernatremia, acidemia, MODS, toxicity

## Abstract

**Objective:**

This case report describes a successful outcome in the treatment of a patient with joint supplement toxicity, which resulted in seizures, severe acidemia, hyperglycemia, hypernatremia, and multiple organ dysfunction. Previous case reports have been published, but this patient presented with different clinical signs and had additional biochemical abnormalities. Treatment modalities varied in this case report, and the patient was discharged sooner than those mentioned in previous reports.

**Case summary:**

A 9-year-old spayed female Maltese mix was presented to a specialty hospital for joint supplement toxicity. Presenting clinical signs were vomiting and seizures. Primary biochemical abnormalities consisted of hypernatremia, hyperglycemia, acidemia, azotemia, and elevated liver enzymes. Treatment involved lowering the sodium quickly given the neurologic signs on presentation. Other treatment modalities consisted of sodium bicarbonate, insulin, and liver protectants. The patient responded quickly and was discharged after 3 days in the hospital.

**New or unique information provided:**

This case report is different in that the patient was having seizures and was also hyperglycemic, in addition to the expected abnormalities of hypernatremia, acidemia, and multiple organ dysfunction. It also differs from prior reports in that the treatment of hypernatremia was managed as an acute process. This case report describes differing clinical signs, biochemical abnormalities, and treatment modalities that may have led to the discharge from the hospital in a shorter time.

## Introduction

Osteoarthritis (OA) is defined as chronic inflammation resulting from the degeneration of the joint cartilage (1). It is a common diagnosis in patients presenting for lameness, affecting 20% of animals older than 1 year (2). It is characterized by varying degrees of lameness, stiffness, chronic pain, loss of mobility, and decreased quality of life. While large breed dogs tend to be overrepresented, all breeds of any age can suffer from OA (2). There are breed predilections, such as Labrador Retrievers and German Shepherds (3). It tends to develop more in patients that are overweight or with underlying metabolic conditions such as diabetes (1). Patients can also develop OA secondary to previous trauma of the joints or abnormal wear on the joint such as in dysplasias or luxations (1).

There is no cure for OA, and treatment is aimed at controlling clinical signs. There are five principles of medical management, namely, body weight management, nutritional supplementation, exercise moderation, physical therapy, and anti-inflammatory medications, which mostly include non-steroidal anti-inflammatories (NSAIDS) (4, 5). While NSAIDs are the golden standard for treatment, there can be side effects such as gastrointestinal upset or ulceration, development of biochemical abnormalities, and acute kidney injury (6). NSAIDS act to control pain and inflammation but do not prevent joint cartilage degeneration. This is where the use of joint supplements has been introduced as a treatment modality.

Given that supplements are considered a cornerstone in the treatment of OA, veterinarians often recommend various nutraceuticals to clients (2). There are numerous supplement ingredients including glucosamine, chondroitin, milk protein, pentose polysulfate, curcumin, manganese, selenium, avocado/soybean unsaponifiables, and polyunsaturated fatty acids, just to name a few (2, 7). The two most common supplements are glucosamine and chondroitin (8). Glucosamine has mild anti-inflammatory effects and contributes to the synthesis of collagen, proteoglycans, and glycosaminoglycans (2). It also has antioxidant and anti-inflammatory mechanisms (9). Chondroitin acts to decrease destructive enzymes in the joint (2). It improves the function and mobility of the joint by improving water retention of the cartilage and increasing the viscosity of the synovial fluid (9).

Joint supplement toxicity has rarely been reported in the veterinary literature. Of the reports published, the main clinical signs are gastrointestinal in nature (10). The main clinicopathological findings are indicative of hepatotoxicity, acute renal lesions, and occasionally electrolyte imbalances. Prognosis has been reported as guarded to poor in those with advanced clinical signs (11, 12).

## Case description

A 9-year-old spayed female, 5.2-kg, Maltese presented to a satellite emergency clinic after ingesting ~40 Dasuquin chews^1^. Upon returning home, the owner found 10 piles of vomitus along with a Dasuquin bag on the floor. The vomitus consisted of undigested food and Dasuquin chews. The patient presented to the emergency hospital having grand mal seizures. An intravenous catheter was placed, and a dose of Diazepam^2^ was administered (0.5 mg/kg, intravenously [IV]). Maropitant^3^ (Cerenia 1 mg/kg, IV) was administered due to ongoing vomiting. Vital parameters showed a rectal temperature of 37.5°C (99.6°F), a heart rate of 140 beats per minute with poor pulses, and a respiratory rate of 40 respirations per minute. The patient's mucous membrane color was pale and dry, with a prolonged capillary refill time (>2 s). On examination, the patient's level of consciousness was obtunded with a grade I/VI heart murmur and had significant ptyalism. Point of care bloodwork showed a PCV of 60% (reference interval, [RI]: 36–46%), total plasma protein of 100.6 g/L (10.6 g/dL; [RI: 65–80 g/L; 6.5.−8.0 g/dL]), hyperglycemia^4^ of 21.6 mmol/L (388 mg/dL; [RI: 4.1–7.9 mmol/L; 74–143 mg/dL]), and lactate of 2.7 mmol/L (RI: 0–2 mmol/L). Full blood work was performed that revealed a hematocrit of 75.3%, (RI: 38.3–56.5%), azotemia with a BUN of 12.3 mmol/L (34.5mg/dL; [RI: 5.3–11.4 mmol/L; 15–32 mg/dL]), creatinine of 123 umol/L (1.4 mg/dL; [RI: 35-123 umol/L; 0.4–1.4 mg/dL]), blood glucose (BG) of >33.3 mmol/L (>600 mg/dL, [RI: 4.1–7.9 mmol/L; 74–143 mg/dL]), sodium of 160 mmol/L (RI: 140–151 mmol/L), hyperchloremia of 130 mmol/L (RI: 106–127 mmol/L), hyperkalemia of 8.7 mmol/L (RI: 3.5–5.0 mmol/L), and hyperphosphatemia at 2.5 mmol/L (7.9 mg/dL; [RI: 0..6–1.6 mmol/L; 1.9–5.0 mg/dL]). Hepatobiliary enzymes showed a normal alanine transaminase, elevations in aspartate transaminase of 85 U/L (RI: 16–55 U/L), alkaline phosphatase of 185 U/L (RI: 5–160 U/L), gamma-glutamyl transferase of 55 U/L (RI: 5–16 U/L), and elevated total bilirubin at 42.75 umol/L (2.5 mg/dL; [RI: 1.7–13.7 umol/L; 0.1-0.8 mg/dL]). Acid–base status was not obtained at that time. The patient was hypotensive with systolic blood pressure (SBP) *via* Doppler^5^ of 44 mm Hg. Two 20 mL/kg IV boluses of Plasmalyte^6^ (Plyte) were administered with minimal improvement in SBP (56, 50 mm Hg). The patient had two additional seizures, so diazepam (0.5 mg/kg IV) was administered again. A single dose of regular insulin^7^ (Humulin-R 0.2 U, IV) was given for persistent hyperglycemia. A recheck of the BG reading was 29 mmol/L (528 mg/dL; [RI: 4.1-7.9 mmol/L; 74–143 mg/dL]), 2 h after regular insulin administration. A loading dose of 140 mg/kg of N-Acetylcysteine^8^ was given IV. On repeat assessment, the patient was hypothermic (31.4 C [93.5° F]) and remained hypotensive (SBP 70 mm Hg). Due to the critical nature, patient care was transferred to the critical care service at the main facility.

On presentation to the main facility (3 h post-treatment initiation), the patient was obtunded with delayed pupillary light and palpebral reflexes and absent menace and gag reflexes. There was slight tachypnea with normal lung sounds. Venous blood gas^9^ (see Table 1) revealed worsening azotemia with a creatinine of 152 umol/L (1.73 mg/dL; [RI: 35–123 umol/L; 0.4–1.4 mg/dL]), persistent hyperglycemia at 36 mmol/L (654 mg/dL; [RI: 4.1–7.9 mmol/L; 74–143 mg/dL]), ongoing hypernatremia (161 mmol/L; [RI: 140–151 mmol/L]), hyperchloremia (>140 mmol/L; [RI: 106–127 mmol/L]), hyperkalemia (5.3 mol; [RI: 3.5–5.0 mmol/L]), and severe acidemia (pH 6.885; [RI: 7.24–7.4]). On abdominal-focused assessment, the stomach was severely distended with fluid, so a nasogastric tube^10^ (NG tube) was placed. Approximately 60 ml of viscous fluid was removed. Oxygen saturation^11^ was normal at 98%, and thoracic radiographs were normal. Aspiration pneumonia with radiographic lag could not be ruled out. The patient was still hypotensive, so two boluses of 20 ml/kg of Plyte were given in addition to a 10-ml/kg bolus of dextrose in water^12^ (D5W). A continuous rate of fluids was started at 2.5 times the resting energy requirement^13^ (RER) with two-thirds of the total volume comprising D5W and one-third Plyte. N-Acetylcysteine was continued at a maintenance dose of 70 mg/kg IV every 6 h. Blood pressures were monitored hourly, and venous blood gases and blood sugar were monitored every 2–4 h. Regular insulin (Humulin R, 1 unit IV) was scheduled to be given if the patient remained hyperglycemic (BG >250 mg/dL). A jugular catheter^14^ (5 French, 13 cm, triple lumen MILA) was placed for ease of blood sampling and to facilitate the administration of multiple fluids and medications. The patient had the area of her neck clipped and aseptically prepared. The patient was measured for the final insertion point of the jugular catheter at the 3–4 intercostal space. A large bore over-the-needle catheter was introduced into the jugular vein, with the tip pointing in the direction of the heart. The guidewire was then inserted into this catheter to the pre-measured length. The needle catheter was removed, and a plastic dilator was used to dilate the vessel. The dilator was then removed, and the catheter was then inserted over the guidewire to the pre-measured length. The catheter was then sutured into place, and a bandage was placed over the catheter. A 6 French urinary catheter^15^ was placed to monitor urine output every 4 h. The patient had the peri vulvar area clipped and aseptically prepared. A stylet was lubricated with sterile lubrication and introduced into the catheter. The tip of the catheter was lubricated with sterile lubrication as well. The urethral papilla was palpated, and the catheter was introduced into the urethra and the bladder. The foley was inflated with the designated amount of sterile saline, and a collection system was attached. NG tube aspiration was performed every 4 h.

**Table 1:** Summary of venous blood gas results.

	**References; Interval**	**Hour 3**	**Hour 5**	**Hour 9**	**Hour 11**	**Hour 13**	**Hour 17**	**Hour 24**	**Hour 36**
pH	7.2–7.4	6.885	6.90	6.97	7.0	7.2	7.17	7.3	7.4
Sodium (meq/L)	140–151	161	166	170	166	160	144	149	151
Potassium (meq/L)	3.5–5.0	5.3	3.9	3.9	2.3	3.8	7.2	4.0	2.8
Chloride (meq/L)	106–127	>140	>140	>140	>140	135	129	125	121
Creatinine (mg/dL)	0.4–1.4	1.73	1.79	1.78	1.43	1.19	1.12	1.10	1.13
Glucose (mg/dL)	74–143	654	608	522	416	190	354	118	133

Another venous blood gas was performed 2 h later (hour 5), which revealed worsening hypernatremia (166 mmol/L; [RI: 140–151 mmol/L]), hyperglycemia (33 mmol/L [608 mg/dL]; [RI: 4.1–7.9 mmol/L; 74–143 mg/dL]), elevated creatinine (153 umol/L [1.79 mg/dL]; [RI: 35–123 umol/L; 0.4–1.4 mg/dL]), and acidemia (pH 6.904; [RI: 7.24–7.4]). Given the worsening hypernatremia, the IV fluids were changed to D5W only and continued at 2.5 RER. The patient was also administered a 10 ml/kg water retention enema. Hypotension persisted, so a broad-spectrum bactericidal antibiotic that acts by inhibiting cell wall synthesis (Unasyn^16^ 30 m/kg, IV) was added for possible gastrointestinal bacterial translocation or undetected aspiration pneumonia. A norepinephrine^17^ continuous rate infusion was started at 1 ug/kg/min. Focal seizures returned, so another dose of diazepam was administered (0.5 mg/kg IV). Venous blood gas showed (hour 9) worsening hypernatremia (170 mmol/L; [RI: 140–151 mmol/L]), hyperglycemia (29 mmol/L; [522 mg/dL]; [RI: 4.1–7.9 mmol/L; 74–143 mg/dL]), and acidemia (pH 6.975; [RI: 7.24–7.4]). Humulin R was given (1 unit, IV). The free water deficit^18^ (13) was calculated, and the volume was given over 12 h utilizing D5W. Plyte was restarted at maintenance RER. The base deficit^19^ (13) was calculated, and a dose of sodium bicarbonate^20^ was given at 1/3 of the calculated deficit (12 mEq, IV). The dose of Humulin R was increased (2 units, IV). After 2 h of the sodium bicarbonate administration, another venous blood gas was performed (hour 11), which revealed a small improvement in the hypernatremia (166 mmol/L; [RI: 140–151 mmol/L]), ongoing hyperglycemia (23 mmol/L; [416 mg/dL]; [RI: 4.1–7.9 mmol/L; 74–143 mg/dL]), improved acidemia (pH 7.096; [RI: 7.24–7.4]), and new hypokalemia (2.3mmol/L; [RI: 3.5–5.0 mmol/L]). Plyte was supplemented with potassium chloride^21^ at 0.07 mEq KCL/kg/hr. A second dose of sodium bicarbonate (12 mEq, IV) was given for persistent acidemia. Venous blood gas was performed (hour 13), which revealed an improvement in the hypernatremia (160 mmol/L; [RI: 140–151 mmol/L]) and acidemia (pH 7.202; [RI: 7.24–7.4]). The BG was normal. Fluids were unchanged with D5W and continued to correct the free water deficit and Plyte at RER with potassium chloride supplementation. The norepinephrine infusion was discontinued as the patient had remained normotensive for the preceding 4 h. By the next venous blood gas (hour 17), the sodium was normalized (144 mmol/L; [RI: 140–151 mmol/L]), but the acidemia worsened slightly (pH 7.177; [RI: 7.24–7.4]). Hyperkalemia returned (7.2 mmol/L; [RI: 3.5–5.0 mmol/L]) as did the hyperglycemia (19.7 mmol/L; [354 mg/dL]; [RI: 4.1–7.9 mmol/L; 74–143 mg/dL]). Humulin R was given at a lower dose (1 unit IV), and the D5W and potassium supplementation were discontinued. Plyte was continued at three times the RER due to high rates of residual gastric volume (average 4.2 ml/kg/h) and polyuria (average 7.8 ml/kg/h).

By day 2 of hospitalization (24 h post-treatment initiation), the patient was persistently normoglycemic and normotensive. Mentation was normal with normal cranial nerve and intact vision. The patient started eating well. Venous blood gas (hour 24) revealed normal sodium, potassium, BG, and only mild acidemia (pH 7.309; [RI: 7.24–7.4]). Plyte was slowly tapered to RER. The residual gastric volume started to decrease, so the NG tube was removed. Urine output decreased as IV fluids were decreased, so the urinary catheter was removed. Regular insulin administration was discontinued. A recheck of venous blood gas in the afternoon revealed hypokalemia (2.8 mmol/L; [RI: 3.5–5.0 mmol/L]) as the only abnormality. Potassium supplementation was added to IV fluids (0.1 mEq KCL/kg/hr). The patient continued to eat and drink with no further seizures.

On day 3 of hospitalization, a chemistry panel revealed elevations in aspartate transaminase (191 U/L; [RI: 16–35 U/L]), alanine transaminase (138 U/L; [RI: 14–87 U/L]), and alkaline phosphatase (244 U/L; [RI: 20–157 U/L]). Other biochemical abnormalities included hypoalbuminemia (24 g/L [2.4 g/dL]; [RI: 23–39 g/dL; 2.3–3.9 mg/dL]), elevated pancreatic serum lipase (380 U/L; [RI: 24–140 U/L]), and elevated creatine phosphokinase (15,796 IU/L; [RI: 59–895 U/L]). The patient was discharged on a liver protectant.

A recheck of bloodwork on day 7 post-discharge was normal. No medications were continued. There was no further follow-up.

## Discussion

In veterinary medicine, reports of joint supplement toxicity are rare. From 2008 to 2009, the Animal Poison Control Center^22^ reported 21 cases of hepatotoxicity, which were reported secondary to ingestion of joint supplements (10). Doses of glucosamine ranged from 183 to 6,667 mg/kg and 45 to 4,000 mg/kg of methylsulfonylmethane (MSM). Information on chondroitin was not reported. Clinical signs were largely gastrointestinal in nature, which developed 30 min to 2 days post-ingestion. Postmortem examination of one patient revealed centrilobular necrosis, acute tubular necrosis, vascular thrombosis, and necrosis of the pancreas and myocardium. In one case report, a 5-year-old spayed female Pug presented for vomiting and ataxia after ingesting ~100 joint supplement tablets. The doses ingested were 10,344 mg/kg of glucosamine, 4,310 mg/kg of MSM, and 5,172 mg/kg of sodium chondroitin sulfate. The patient presented obtunded with hypernatremia, hyperchloremia, and acidosis. The dog in this case also had elevations in liver enzymes and clotting times which progressed to hepatic failure. The patient was euthanized on day 6. The postmortem revealed centrilobular liver necrosis, acute tubular necrosis, renal thrombosis, and necrosis of the pancreas and myocardium. This patient also had increased manganese concentrations in the liver and kidney (14). In a second case report, a 5-year-old spayed female Bernese Mountain dog presented with vomiting and melena after ingesting over 200 joint supplements^23^, with ingestion of up to 2,173 mg/kg of glucosamine and 217 mg/kg of chondroitin sulfate. The patient had elevated liver enzymes, a prolonged prothrombin time, and developed progressive hyperbilirubinemia and azotemia during hospitalization. This patient did not develop hypernatremia or hyperglycemia, and acid–base status was not assessed. The patient was euthanized on day 5 due to worsening clinical status and bloodwork changes. Postmortem examination had similar findings to previous cases with centrilobular liver necrosis, acute tubular necrosis, renal thrombosis, and necrosis of the pancreas and myocardium (11). The most recently published report of joint supplement toxicity was a 6-year-old neutered male Dachshund that presented after ingesting an unknown quantity of Dasuquin joint supplements. Potential exposure doses were 1,900 mg/kg of glucosamine, 750 mg/kg of chondroitin sulfate, and 870 mg/kg of MSM. The patient suffered from neurological deficits, prolonged clotting times, thrombocytopenia, hepatotoxicity, azotemia, hypernatremia, and severe metabolic acidosis. The hypernatremia and acid–base abnormalities resolved by day 5, and the patient was discharged on day 10 (12).

In this case, the patient ingested 40 Dasuquin chews. Doses ingested were 4,615 mg/kg of glucosamine hydrochloride and 1,923 mg/kg of sodium chondroitin sulfate. This dose of glucosamine falls within the ranges as reported by the Animal Poison Control Center (10), and both the glucosamine and chondroitin doses were higher than the exposure doses of the previous two case studies in which one lived and one was euthanized. The complete composition of the Dasuquin chews is presented in the footnotes for reference.

The patient presented after having multiple seizures, with no prior history of seizures. Since the patient presented with a comatose mentation, additional GI decontamination such as the administration of activated charcoal was not considered safe at the time. While prior reports listed ataxia and mentation changes as clinical signs, no other patients had seizures. The seizures likely occurred secondary to the acute nature of the hypernatremia causing water to osmotically flow out of brain cells, decreasing the brain volume rapidly which causes rupture of cerebral vessels (13). The cause of the hypernatremia, which has been reported in other case reports, is likely multi-factorial. This patient was experiencing vomiting leading to hypotonic fluid losses and was also hypotensive leading to activation of the renin-angiotensin-aldosterone system that acts to retain sodium (13). The administration of sodium bicarbonate likely contributed to the initial rise in sodium levels after hospitalization (albeit mild). The Dasuquin had sodium chondroitin sulfate as one of the primary ingredients. It is possible that the salt formulation of this ingredient may have contributed to the hypernatremia but this is not a consistent finding in toxicity cases. Chondroitin also has an osmotic pull as its mechanism of action (9). It is also possible that an overdose caused excessive fluid to be pulled into the gastrointestinal tract, causing further fluid depletion of the extracellular space. Since the hypernatremia was assumed to be an acute process, supported by the seizures' mental state, the sodium normalized within 24 h. This is different from the previous case report in which the hypernatremia persisted for several days and the patient was not discharged until day 10.

The patient also had a mild degree of azotemia that resolved after the first day of therapy. It is suspected that the azotemia was secondary to dehydration or a pre-renal component since the azotemia resolved with aggressive fluid therapy, but renal damage from the Dasuquin chews could not be completely excluded. Other studies documented case reports of joint supplement toxicity which included acute tubular necrosis, but the damage was minimal in comparison to the hepatic damage from the joint supplements (11, 12, 14).

The exact etiology of hepatic damage with joint supplements is unknown. Nobles et al. proposed several theories for hepatic toxicity which included heavy metal contamination, synergistic effects of multiple ingredients, toxic metabolite formulation, and misinformation on the levels (11). This patient also had elevated hepatobiliary enzymes, but the degree of liver enzyme elevation was not as severe as the other case reports. Since the degree of liver enzyme elevation was minor in comparison to other reports, it was assumed that the damage was also minor, so additional tests for liver failure such as clotting times and ammonia levels were not performed. However, the dose of ingested glucosamine and chondroitin was similar, if not more, when compared with other reported toxicities. Therefore, dose-dependent toxicity may not be the case with glucosamine and chondroitin, leading one to believe other mechanisms of toxicity may be at play. This patient received acetylcysteine while in the hospital to mitigate hepatotoxicity. However, it is important to note that this patient was discharged on day 3 and that liver enzymes may have continued to increase prior to returning to normal on day 7.

This patient was administered sodium bicarbonate given the severe acidosis. The decision to administer this medication was not taken lightly. As previously noted, this may have contributed to the patient's hypernatremia. The administration of sodium bicarbonate can also cause a paradoxical acidosis of the cerebrospinal fluid (15). This is secondary to the diminished respiratory drive and a rise in the partial pressure of carbon dioxide, which readily crosses the blood–brain barrier, augmenting CSF acidosis (15). In a study conducted with an induced metabolic acidosis of calves, the administration of sodium bicarbonate to correct acidosis rapidly (over 4 h) or gradually (over 24 h) did not result in a significant decrease in CSF pH (15). However, it is also known that severe acidosis can decrease cardiac output, myocardial contractility, and renal and hepatic blood flow and cause hypotension that is refractory to vasopressors (16). Acidosis can also impair lactate clearance, which further perpetuates acidosis (16). Acidosis also causes insulin resistance leading to persistent hyperglycemia. Therefore, in this case, it was felt that the administration of sodium bicarbonate outweighed the potential risks.

Different from other cases, this patient was also profoundly hyperglycemic and was initially refractory to insulin administration. Glucosamine has been studied in humans and given in excess in non-diabetic patients, and the baseline glucose concentration only increased mildly and did not cause insulin resistance (17). The hyperglycemia may have been preset due to the critical nature of the patient. Critical illness has been shown to cause hyperglycemia due to a combination of increased secretion of catabolic hormones, increased hepatic gluconeogenesis, and resistance to the peripheral and hepatic actions of insulin (18). The norepinephrine infusion may have also contributed to hyperglycemia. Previous case reports showed evidence of pancreatitis and pancreatic thrombi on histopathology. It is possible that this patient developed acute pancreatitis leading to hyperglycemia. However, this patient had a sudden resolution of hyperglycemia which is not always typical of pancreatitis. The patient was administered D5W to correct the free water deficit which may have contributed to the hyperglycemia while in hospital. The hyperglycemia became responsive to insulin as the acidosis improved, and the D5W was tapered and stopped. The hyperglycemia was resolved, with no insulin requirement, by day 3. The hyperglycemia was also noted to be vastly different on the handheld glucometer versus the in-house chemistry machine when the patient was first admitted to the main facility. This is likely attributed to the patient's severe hemoconcentration, which resulted in lower plasma blood glucose readings on the handheld glucometer in comparison to a chemistry analyzer (19).

This case report describes a successful outcome in the treatment of a patient with joint supplement toxicity which resulted in expected abnormalities of hypernatremia, acidemia, and multiple organ dysfunction. In similar case reports, the hypernatremia was corrected slower and resulted in a longer hospitalization stay. However, this case differed in that the patient also presented with seizures and hyperglycemia. Treatment of this patient differed in that the hypernatremia was treated as an acute process and required insulin therapy. Despite the critical nature and previously reported guarded-to-poor prognosis, this patient was discharged after only 3 days of therapy with no long-term biochemical abnormalities. As the owner of the patient approved all the treatments and no novel therapies were used, ethical approval was not required in this study, and the owner consented to all the treatments.

## Data availability statement

The raw data supporting the conclusions of this article will be made available by the authors, without undue reservation.

## Ethics statement

Ethical review and approval was not required for the study on animals in accordance with the local legislation and institutional requirements. Written informed consent was obtained from the owners for the participation of their animals in this study. Written informed consent was obtained from the participant/patient(s) for the publication of this case report.

## Author contributions

All authors listed have made a substantial, direct, and intellectual contribution to the work and approved it for publication.

## Footnotes

^1^Dasuquin Chews Soft Chews with MSM, Nutramax Laboratories, Veterinary Sciences Inc. DESCRIPTION: Each soft chew contains glucosamine hydrochloride 99% 600 mg, methylsulfonylmethane (MSM) 400 mg, sodium chondroitin sulfate 250 mg, avocado/soybean unsaponifiable (ASU) powder 45 mg, Boswellia serrata extract 20 mg, green tea extract 16mg. Other ingredients: glycerin, lecithin, mixed tocopherols (preservative), natural flavor, rosemary extract, silicon dioxide, soy protein isolate, starch, and vegetable oil.

^2^Diazepam 5mg/ml, Hospira Inc, Lake Forest, IL. DESCRIPTION: Diazepam is a benzodiazepine derivative. Diazepam Injection, USP is a sterile solution, and each ml contains 5 mg diazepam compounded with 40% propylene glycol, 10% alcohol, 5% sodium benzoate and benzoic acid as buffers, and 1.5% benzyl alcohol as a preservative.

^3^Maropitant, Zoetis LLC, Kalamazoo, MI. DESCRIPTION: Maropitant is a neurokinin (NK1) receptor antagonist that blocks the pharmacological action of substance P in the central nervous system. Maropitant is the non-propriety designation for a substituted quinuclidine. Each ml of CERENIA injectable solution contains 10 mg maropitant, 63 mg sulphobutylether-beta-cyclodextrin, and 3.3 mg mega-cresol and water for injection.

^4^Vet Glucogauge, Covetrus, Overland Mark, KS.

^5^Doppler, Parks Medical Electronics Inc, Aloha, OR.

^6^Plasmalyte, Baxter Healthcare Corporation, Deerfield, IL. DESCRIPTION: Each 100 ml contains: 526 mg sodium chloride, USP; 502 mg sodium gluconate, USP; 368 mg sodium acetate trihydrate, USP; 37 mg potassium chloride, USP; 30mg magnesium chloride, USP; pH adjusted with sodium hydroxide. Electrolytes per 1,000 ml: sodium 140mEq; potassium 5mEq; magnesium 3mEq; chloride 98mEq; acetate 27mEq; gluconate 23mEq. Total osmolar concentration: 294mOsmol/L (calculated). pH 7.4.

^7^Humulin R, Lilly USA, Indianapolis, IN. DESCRIPTION: HUMULIN R (insulin human) injection is a short-acting human insulin for subcutaneous or intravenous use. Human insulin is produced by recombinant DNA technology, utilizing a non-pathogenic laboratory strain of *Escherichia coli*. HUMULIN R for subcutaneous or intravenous use is a sterile, clear, aqueous, and colorless solution that contains human insulin 100units/ml, glycerol 16mg/ml, metacresol 2.5mg/ml, endogenous zinc (approximately 0.015mg/100 units), and water for injection.

^8^20% N-Acetylcysteine, Hospira Inc., Lake Forest, IL. DESCRIPTION: Acetylcysteine solution is for inhalation (mucolytic agent) or oral administration (acetaminophen antidote) and is available as a sterile, unpreserved solution (not for injection). Acetylcysteine is the N-acetyl derivative of the naturally occurring amino acid, L-cysteine. Chemically, it is N-acetyl-L-cysteine. Each ml of the 20% solution contains acetylcysteine 200 mg, edetate disodium, and dihydrate 0.5mg. The solutions also contain sodium hydroxide and may contain hydrochloric acid for pH adjustment.

^9^EPOC blood gas analyzer, Woodbridge, NY.

^10^Nasogastric tube, MILA International Inc, Florence, KY.

^11^PalmsSAT 2500, Plymouth, MN.

^12^Dextrose in water, Hospira Inc, Lake Forest, IL. DESCRIPTION: 5% Dextrose Injection, USP solution is sterile and non-pyrogenic. It is a parenteral solution containing dextrose in water for injection intended for intravenous administration. Each 100ml contains dextrose hydrous 5g in water for injection. The caloric value is 170 kCal/L. 252mOsmol/L (calculated). pH 4.3.

^13^Resting Energy Requirement: 70 x body weight in kilograms to the $\frac{3}{4}$ power.

^14^Triple lumen jugular catheter, MILA International Inc, Florence, KY.

^15^Foley urinary catheter, MILA International Inc, Florence, KY.

^16^Unasyn, Roerig (Pfizer), New York, NY. DESCRIPTION: UNASYN is an injectable antibacterial combination consisting of the semi-synthetic antibacterial ampicillin sodium and the beta-lactamase inhibitor sulbactam sodium for intravenous or intramuscular administration. Ampicillin sodium is derived from the penicillin nucleus and 6-aminopenicillanic acid. Sulbactam sodium is a derivative of the basic penicillin nucleus. 1.5 g of UNASYN (1 g of ampicillin as the sodium salt plus 0.5 g of sulbactam as the sodium salt) parenteral contains approximately 115 mg (5 mEq) of sodium.

^17^Norepinephrine, Teva Pharmaceuticals USA, Inc, Parsippany, NJ. DESCRIPTION: Norepinephrine (sometimes referred to as l-arterenol/Levarterenol or l-norepinephrine) is a sympathomimetic amine that differs from epinephrine due to the absence of a methyl group on the nitrogen atom. Norepinephrine is supplied in a sterile aqueous solution in the form of bitrate salt to be administered by intravenous infusion following dilution. Norepinephrine is sparingly soluble in water, very soluble in alcohol and ether, and readily soluble in acids. Each ml contains the equivalent of 1 mg base of norepinephrine and sodium chloride for isotonicity.

^18^Free Water Deficit calculation: Wt (kg) x [Na (presentation)/Na (normal)].

^19^Base Deficit calculation: (0.3 x Wt (kg) x [normal HCO3 – current HCO3]).

^20^Sodium Bicarbonate 8.4%, Hospira Inc., Lake Forest, IL. DESCRIPTION: Sodium Bicarbonate Injection, USP is a sterile, non-pyrogenic, hypertonic solution of sodium bicarbonate (NaHCO3) in water for injection for administration by the intravenous route as an electrolyte replenisher and systemic alkalizer. Sodium bicarbonate, 84mg is equal to 1mEq each of Na and HCO3.

^21^Potassium chloride, Hospira Inc, Lake Forest, IL. DESCRIPTION: Potassium Chloride for Injection Concentrate, USP is a sterile, non-pyrogenic, concentrated solution of potassium chloride. USP in water for injection is administered by intravenous infusion only after dilution in a larger volume of fluid. The solutions contain no bacteriostat, antimicrobial agent, or added buffer (except for pH adjustment), and each is intended only for single-dose injection (after dilution).

^22^Animal Poison Control Center, American Society for the Prevention of Cruelty to Animals, New York, NY.

^23^Joint MAX triple strength, Pet Health Solutions, Union City, CA. DESCRIPTION: Active ingredients per two soft chews: glucosamine HCl (shellfish source) 1,000 mg, methylsulphonylmethane (MSM) 600 mg, creatine monohydrate 400 mg, and chondroitin sulfate (porcine source) 150 mg; Propriety antioxidant blend: Brewer's yeast, cinnamon, turmeric root powder, grape seed extract, valerian root, cranberry juice extract 150 mg, eicosapentaenoic acid (EPA) 132 mg, docosahexaenoic acid (DHA) 88 mg, vitamin C 50 mg, vitamin E 50IU, citrus bioflavonoids 10 mg, hyaluronic acid 2 mg, L-glutathione 2 mg, zinc 2 mg, alpha lipoic acid 400 mcg, and selenium 2 mcg. Inactive ingredients: dicalcium phosphate, glycerine, magnesium stearate, marine lipid concentrates, molasses, polyethylene oxide, powdered liver (non-bovine), starch, sucrose, TBHQ, vegetable fiber, vegetable oil, and vegetable shortening.

## References

[1] Musco N Vassalotti G Mastellone V. Effects of a nutritional supplement in dogs affected by osteoarthritis. Vet Med Sci. (2019) 5:325–35. 10.1002/vms3.182 31313893 PMC6682793

[2] Bhathal A Spryszak M Louizos C Frankel G. Glucosamine and chondroitin use in canines for osteoarthritis: a review. Open Vet J. (2017) *7*:36–49. 10.4314/ovj.v7i1.6 28331832 PMC5356289

[3] Anderson K O'Neill D Brodbelt D. Prevalence, duration, and risk factors for appendicular osteoarthritis in a UK dog population under primary veterinary care. Sci Rep. (2018) 8:5641. 10.1038/s41598-018-23940-z 29618832 PMC5884849

[4] Beale BS. Use of nutraceuticals and chondroprotectants in osteoarthritic dogs and cats. Vet Clin North Am Small Anim Pract. (2004) 34:271–89. 10.1016/j.cvsm.2003.09.008 15032132

[5] Fossum TW. Small Animal Surgery. 5^th^ ed. Philadelphia: Elsevier. (2019) p. 105–117.

[6] Buttgereit F Burmester GR Simon LS. Gastrointestinal toxic side effects of nonsteroidal anti-inflammatory drugs and cyclooxygenase-2–specific inhibitors. Am J Med. (2001) 110:13–29. 10.1016/S0002-9343(00)00728-2 11173045

[7] Comblain F Serisier S Bathelemy N Balligand M Henrotin Y. Review of dietary supplements for the management of osteoarthritis in dogs in studies from 2004-2014. J Vet Pharmacol Ther. (2016) 30:1–15. 10.1111/jvp.12251 26205697

[8] Rychel JK. Diagnosis and treatment of osteoarthritis. Top Companion Anim Med. (2010) 25:20–5. 10.1053/j.tcam.2009.10.005 20188335

[9] Jerosch J. Effects of glucosamine and chondroitin sulfate on cartilage metabolism in OA: outlook on other nutrient partners especially omega- 3 fatty acids. Int J Rheumatol. (2011) 969012:17. 10.1155/2011/969012 21826146 PMC3150191

[10] Peter KM. Veterinarians as leaders in animal welfare. J Am Vet Med Assoc. (2010) 236:509–10. 10.2460/javma.236.5.509 20187811

[11] Nobles I Safdar K. Multiorgan dysfunction syndrome secondary to joint supplement overdose in a dog. Can Vet J. (2015) 56:361–4. 25829554 PMC4357907

[12] Weatherson HO Bellis T Tse Y. The successful treatment of multiple organ dysfunction syndrome and severe hypernatremia, secondary to joint supplement toxicity in a dog. J Vet Emerg Crit Care. (2021) 31:432–8. 10.1111/vec.13033 33751791

[13] Dibartola SP. Fluid, Electrolytes and Acid-Base Disorders in Small Animal Practice. 4^th^ ed. St. Louis: Saunders/Elsevier. (2012) p. 80–91, 254-286. 10.1016/B978-1-4377-0654-3.00017-2

[14] Borchers A Epstein S Gindiciosi B. Acute enteral manganese intoxication with hepatic failure due to ingestion of a joint supplement overdose. J Vet Diagn Invest. (2014) 26:658–63. 10.1177/1040638714544316 25080444

[15] Abeysekara S Zello G Lohmann K. Infusion of sodium bicarbonate in experimentally induced metabolic acidosis does not provoke cerebrospinal fluids (CSF) acidosis in calves. Can J Vet Res. (2012) 76:16–22. 22754090 PMC3244283

[16] Drobatz K Hopper K Rozanski E Silverstein D. Textbook of Small Animal Emergency Medicine. 2^nd^ ed. New York: John Wiley & Sons. (2018) p. 690–699. 10.1002/9781119028994

[17] Saghafi M Karimi M Bonakdaran S Massoudi N. Oral glucosamine effect on blood glucose and insulin levels in patients with no0n-diabetic osteoarthritis: a double-blind, placebo-controlled clinical trial. Arch Rheumatol. (2016) 31:340–5. 10.5606/ArchRheumatol.2016.5632 30375553 PMC6190977

[18] Brealey D Singer M. Hyperglycemia in critical illness: a review. J Diabetes Sci Technol. (2009) 3:1250–60. 10.1177/193229680900300604 20144378 PMC2787024

[19] Cutler D Koeing A DiGirolamo N Mayer J. Investigation for correction formulas on the basis of packed cell volume for blood glucose concentration measurements obtained with portable glucometers when used in rabbits. Am J Vet Res. (2020) 81:642–50 10.2460/ajvr.81.8.642 32700996

